# Cognitive performance in systemic lupus erythematosus patients: a cross-sectional and longitudinal study

**DOI:** 10.1186/s41927-022-00253-3

**Published:** 2022-04-20

**Authors:** L. Langensee, J. Mårtensson, A. Jönsen, K. Zervides, A. Bengtsson, J. Nystedt, B. Cannerfelt, P. Nilsson, P. Mannfolk, J. Lätt, T. Rumetshofer, P. C. Sundgren

**Affiliations:** 1 Department of Clinical Sciences Lund, Logopedics, Phoniatrics and Audiology, Faculty of Medicine, Lund University, 22185 Lund, Sweden; 2 Department of Clinical Sciences Lund, Rheumatology, Faculty of Medicine, Lund University, 22185 Lund, Sweden; 3 Department of Clinical Sciences Lund, Neurology, Faculty of Medicine, Lund University, 22185 Lund, Sweden; 4 Department of Clinical Sciences Lund, Radiology, Faculty of Medicine, Lund University, 22185 Lund, Sweden; 5 Department of Medical Imaging and Physiology, Skåne University Hospital, 221 85 Lund, Sweden; 6 Lund University BioImaging Center (LBIC), Lund University, 22185 Lund, Sweden

**Keywords:** SLE, NPSLE, Lupus, Cognitive impairment, Neurocognitive testing, Cognition, CNS-VS

## Abstract

**Background:**

Previous research has provided evidence for cognitive dysfunction as a common symptom of systemic lupus erythematosus (SLE). In light of this, the primary goal of this study was to investigate how cognitive impairment in this patient group develops over time. In addition, the present dataset contributes to delineating the specific abilities that are impaired in SLE patients as well as answering the question whether the disease affects the cognition of SLE patients with neuropsychiatric manifestations (NPSLE) and without (non-NPSLE) in distinct ways.

**Methods:**

91 female participants (33 NPSLE, 29 non-NPSLE, 29 healthy controls (HC)) underwent standardized neurocognitive testing. A total of ten different cognitive abilities were assessed, among others executive function, memory, and attention. Some of the participants (30 NPSLE patients, 22 non-NPSLE, 13 HC) were tested twice (mean time between testing sessions: 50 months) to enable longitudinal tracking of cognitive abilities. Analyses of Variance (ANOVA) were conducted to determine whether cognitive performance differed cross-sectionally between the groups. Linear mixed effects models were fit to investigate performance differences between the groups over time.

**Results:**

Cross-sectional analysis at follow-up demonstrated that the cognitive performance of both NPSLE and non-NPSLE was significantly lower than that of HC for the *motor speed* and the *psychomotor speed* domain. Additionally, NPSLE patients performed significantly weaker than HC in the *complex attention* domain. At the same time, the cross-sectional data did not yield any support for performance differences between NPSLE and non-NPSLE patients. Weak positive correlations between disease duration and *psychomotor speed*, *motor speed* and *reaction time* emerged. A temporal progression of cognitive dysfunction in SLE patients was not confirmed.

**Conclusions:**

Cognitive performance is affected in both non-NPSLE and NPSLE patients. However, a linear decline in performance over time could not be verified. More in-depth longitudinal assessments of cognition in SLE patients are needed to establish how cognitive abilities in this patient population develop over time.

**Supplementary Information:**

The online version contains supplementary material available at 10.1186/s41927-022-00253-3.

## Background

The etiology of systemic lupus erythematosus (SLE) is thought to be multi-factorial with genetic, hormonal, immune and environmental factors contributing to the onset of the disease [1–3]. As a result, various parts and organs of the body can be affected, ranging from the skin, joints, kidneys, heart and lungs to the central and peripheral nervous system [4]. Among the many symptoms that SLE patients can experience, problems with cognitive functioning are frequently reported [5, 6]. In 1999, the American College of Rheumatology (ACR) published case definitions for 19 different syndromes associated with neuropsychiatric SLE, one of which is cognitive dysfunction [7]. Neuropsychiatric involvement is common in SLE patients and cognitive dysfunction was shown to be among the three most frequent manifestations of these [8]. Cognitive dysfunction affects SLE patients both with (NPSLE) and without (non-NPSLE) overt central nervous system involvement and is associated with negative effects on quality of life [5, 9–12]. Impairment in executive function is a hallmark of cognitive dysfunction in SLE; other frequently affected domains include attention, memory, language and visuospatial processes [5, 13].

Several different neuropsychological tools have been used in the past to assess cognitive dysfunction in SLE [5]. Aside from traditional paper–pencil based tests, several computer-based tools are available for assessing cognition, for example the ANAM (Automated neuropsychological assessment metrics) [14], the CANTAB (Cambridge Neuropsychological Test Automated Battery) [15], and the CNS-VS (CNS-Vital Signs) [16]. All of these have previously been used in SLE research; the ANAM more frequently [17–20] than the CANTAB and the CNS-VS [21–24]. However, the cognitive domains that CNS-VS assesses, match the abilities that have previously been shown to be affected in SLE patients and the different subtests are well-established measures within neuropsychological evaluation, which is the reason why it was decided to use CNS-VS for the current study. CNS-VS has also previously been used to assess cognitive impairment in brain tumors [25, 26], and tested and validated in patients with traumatic brain injury, dementia and MS [27–29].

While a number of studies have addressed the cognitive functioning of SLE patients, research on the longitudinal course of cognitive impairment in this population and potential fluctuations over time is still limited [30–36]. The existing literature suffers from limitations, such as small sample size, lack of proper controls and/or of patients without any history of neuropsychiatric involvement as well as unclear description of the patient cohort, which makes interpreting the results and drawing comparisons between them challenging. An additional complication is the wide variety of neurocognitive testing methods used, ranging from traditional pencil and paper form to computer-based examinations. Because of the above, existent findings are to an extent inconsistent and hence difficult to sum up. Some suggest that more years of education and longer disease duration are associated with a lower risk of cognitive impairment [6]. Some found cognitive dysfunction to be uncorrelated with age [17] or to accumulate over time [34, 37], while others indicate that it might improve in those whose psychiatric disorders have resolved [38]. Others found cognitive impairment to not change over time [32].

To help remedy the shortage of longitudinal studies on cognitive dysfunction in SLE patients, a follow-up assessment of cognitive performance in a sample of previously examined SLE patients was performed, including patients both with and without neuropsychiatric involvement, as well as healthy controls. The participants underwent the same computerized neurocognitive examination on two occasions, providing data to help us evaluate both cross-sectional and longitudinal patterns in the cognitive performance of both non-NPSLE and NPSLE patients.

## Materials and methods

A longitudinal, prospective study, with baseline and follow-up conducted with the same set-up, included a total of 91 right-handed, female participants. Out of these, 62 were outpatients with SLE and 29 were healthy controls (HC; age range 24–55, median 35.0 years). Both SLE patients with and without neuropsychiatric involvement were included. Following the baseline assessment, participants were asked to return for a follow-up, which a subset of 52 SLE patients, including 30 NPSLE and 22 non-NPSLE, and 13 HC (age range 25–52 at baseline, median age at initial study 41.0 years and at follow-up 44.0 years) agreed to. Information about the age of the patients at the different time points can be found in Tables 1 and 2 (additional details regarding age can be found in the Additional file 1). As SLE is far more common in women, males were excluded. Only right-handed individuals were included to reduce the risk of hemispheric differences influencing the Magnetic Resonance Imaging (MRI) portion of the study.

**Table 1:** Overview of questionnaire, and treatment data for the cross-sectional sample

	Non-NPSLE	NPSLE
No. of subjects	29	33
Age range (median)	23–56 (38)	22–55 (44)
Disease duration (years)	17.0 (4.1–26.9)	14.0 (4.0–28.0)
SDI-score	0.0 (0.0–1.6)	1.0 (0.0–4.0)
SLEDAI2k-score	2.0 (0.0–5.2)	0.0 (0.0–9.1)
anti-nuclear antibodies	29 (100%)	31 (93%)
anti-ds-DNA antibodies	18 (62%)	21 (63%)
anti Sm-nuclear antigen	5 (17%)	3 (9%)
Glucocorticoids	18 (62%)	21 (63%)
Daily dose of glucocorticoids	2.0 (0.0–10.0)	4.5 (0.0–10.0)
Antimalarials	26 (89%)	25 (75%)
Cyclophosphamide	0 (0%)	0 (0%)
Azathioprine	2 (6%)	13 (39%)
Cyclosporine A	2 (6%)	0 (0%)
MMF	4 (13%)	6 (18%)
Rituximab	1 (3%)	1 (3%)
IVIG	1 (3%)	1 (3%)
Methotrexate	1 (3%)	3 (9%)
Belimumab	8 (27%)	3 (9%)
Antihypertensive treatment	5 (17%)	9 (27%)
aPL+	5 (17%)	14 (42%)

Data presented as median values (with 95% confidence intervals); respectively absolute number of patients in the sample (with percentage) that received a specific treatment

*SDI* systemic lupus international collaborating/clinical/ACR organ damage index, *SLEDAI-2k* SLE disease activity index 2000, *MMF* mycophenolate mofetil, *IVIG* intravenous immunoglobulin, *aPL* antiphospholipid

**Table 2:** Overview of questionnaire, and treatment data of the patients that participated both at baseline and follow-up

No. of subjects	Baseline		Follow-up	
	Non-NPSLE	NPSLE	Non-NPSLE	NPSLE
	22	30	22	30
Age range (median)	18–51 (34.5)	18–49 (41)	23–56 (38.5)	22–55 (44)
Disease duration (years)	13.0 (1.0–24.0)	10.0 (1.0–24.0)	17.2 (5.1–27.7)	14.6 (4.5–28.1)
SDI-score	0.0 (0.0–1.9)	0.0 (0.0–3.0)	0.0 (0.0–1.9)	1.0 (0.0–4.0)
SLEDAI-2 k-score	1.0 (0.0–4.0)	2.0 (0.0–7.3)	2.0 (0.0–3.9)	2.0 (0.0–10.2)
anti-nuclear antibodies	22 (100%)	29 (96%)	22 (100%)	28 (93%)
anti-ds-DNA antibodies	12 (54%)	18 (60%)	12 (54%)	18 (60%)
anti Sm-nuclear antigen	4 (18%)	4 (13%)	4 (18%)	3 (10%)
Glucocorticoids	17 (77%)	25 (83%)	13 (59%)	19 (63%)
Daily dose of glucocorticoids	4.5 (0.0–14.7)	5.0 (0.0–12.7)	2.0 (0.0–10.0)	5.0 (0.0–10.0)
Antimalarials	20 (90%)	23 (76%)	19 (86%)	22 (73%)
Cyclophosphamide	1 (4%)	0 (0%)	0 (0%)	0 (0%)
Azathioprine	9 (40%)	11 (36%)	2 (9%)	11 (36%)
Cyclosporine A	0 (0%)	0 (0%)	2 (9%)	0 (0%)
MMF	3 (13%)	6 (20%)	2 (9%)	5 (16%)
Rituximab	0 (0%)	0 (0%)	0 (0%)	1 (3%)
IVIG	1 (4%)	1 (3%)	1 (4%)	1 (3%)
Methotrexate	1 (4%)	0 (0%)	1 (4%)	3 (10%)
Belimumab	5 (22%)	2 (6%)	7 (31%)	3 (10%)
Antihypertensive treatment	5 (22%)	9 (30%)	3 (13%)	9 (30%)
aPL+	5 (22%)	13 (45%)		

Data presented as median values (with 95% confidence intervals); respectively absolute number of patients in the sample (with percentage) that received a specific treatment

All SLE patients have been aPL+ in significant titers previously during their disease

*SDI* systemic lupus international collaborating/clinical/ACR organ damage index, *SLEDAI-2k* SLE disease activity index 2000, *MMF* mycophenolate mofetil, *IVIG* intravenous immunoglobulin, *aPL* antiphospholipid

Thirteen HC participating in the initial study were recruited to participate in this follow-up study. In addition, we recruited 16 aged-matched, female, right-handed HC from among health care workers and university employees at our institution. The average time between baseline and follow-up assessment was 50 months (range 19.4–78 months, SD 13.75 months). An overview of clinical, and treatment information can be found in Table 1 (for the cross-sectional sample) and 2 (for the longitudinal sample).

All participants in the study, regardless of whether they were patients or healthy controls, had to meet the following inclusion criteria: female sex, aged 18–55 years, right-handed, able to perform a MRI examination (since the data for this study was collected in the context of a larger project which included MRI scanning), able to give informed consent, and speak Swedish fluently (to be able to perform the neurocognitive testing). Patients were recruited consecutively from the pre-existing SLE cohort at the Clinic of Rheumatology, Lund, Sweden. In addition to the above criteria, they had to have a verified SLE diagnosis and they had to fulfill at least four ACR classification criteria for SLE [39] in order to be included in the study. Additional file 1: Tables of the NPSLE manifestations in the cohort is present.

The HC had to be free from any moderate or major mood-disorders, autoimmune diseases, as well as any previously diagnosed neurological or neuropsychiatric conditions.

### Neurocognitive examination

As in previous work [23] CNS-VS was used, a standardized, computerized neurocognitive test battery. It comprises seven conventional neuropsychological tests: verbal and visual memory, finger tapping, symbol digit coding, the Stroop Test, a test of shifting attention and a continuous performance test. These seven tests cover ten cognitive domains: verbal memory, visual memory, psychomotor speed, reaction time, complex attention, cognitive flexibility, processing speed, executive function, simple (visual) attention, and motor speed [16]. A brief description of the tests and their clinical relevance are summarized in a publication by Rydelius and colleagues [26].

A psychologist, who remained present for the duration of the session, tested participants individually. A brief oral introduction by the psychologist, explaining the testing procedure was given to each participant. Following this, they completed the test battery independently according to the instructions that were given on the screen prior to each of the tests. Additional clarifications were provided when requested. Each of the individual tests had to be performed for a predetermined amount of time and most tests were in turn timed internally, so that a response had to be given within the provided time window. Completing the entire test battery took approximately 30 min (variations occurred due to participants taking varying amounts of time to read the instructions).

### Clinical examination

Patients were assessed by specialists in neurology and rheumatology and individual case discussions were conducted between the two neurologists and the three rheumatologists participating in the study if the initial “attributions” were not concordant to obtain consensus prior to inclusion. Symptoms from the nervous system were attributed to SLE or other causes, taking into account medication, pain, fatigue, co-morbid conditions and prior medical history, by a consensus decision. Patients were categorized as NPSLE or non-NPSLE in compliance with the American College of Rheumatology Case Definitions for NPSLE [7]. Notably, mild cognitive dysfunction in SLE is common and this manifestation can often not be attributed to SLE with certainty, thus also patients in the non-NPSLE group may experience cognitive dysfunction. Organ damage was recorded according to the SLICC/ACR damage index (SLICC/ACR-DI) [40], and disease activity was assessed using the Systemic Lupus Erythematosus Disease Activity Index 2000 (SLEDAI-2K) [41].

### Statistical analysis

All calculations were performed in R version 4.0.3 [42]. Standard scores, derived from a large (1600 +) normative sample provided by the CNS-VS software, were used for the analysis. These scores have a mean of 100 and a standard deviation of 15. The normative sample consists of age-matched, healthy participants free from neurological or psychiatric disorders and any learning disabilities. All values that were more than 1.5 times the interquartile range below the first quartile or more than 1.5 times the interquartile range above the third quartile were considered outliers and were removed from the analysis. Generalized eta square (η^2^_G_) was used as a measure of effect size, where 0.01 indicates a small effect, 0.06 a medium effect and 0.14 a large effect. Whenever multiple comparisons were computed, the resulting p-values were corrected according to the Holm-Bonferroni method [43]. All functions used in the analyses stem from the rstatix or the stats package [44, 45].

### Cross-sectional analysis (at follow-up)

To assess the effect of group (with the three levels HC, NPSLE and non-NPSLE) on mean performance in each of the cognitive domains, ten individual one-way analyses of variance (ANOVA; Type II Sum of squares) were computed. Normal distribution at each factor level was checked with the help of the Shapiro–Wilk test as well as visually by means of Q–Q plots. The data were normally distributed at all factor levels, except for the *simple attention* domain. Since the simple attention domain were non-normally distributed in all three groups, a Kruskal–Wallis test was used for further analysis. Homogeneity of variances was confirmed by means of Levene’s test.

### Longitudinal analysis

For the longitudinal analysis, separate linear mixed effect models were fit to examine the effects of group, time between test sessions, and a possible interaction of the two on performance in each of the ten cognitive domains. The individual test scores on the respective cognitive tests were entered into the model as a dependent variable. Group (with the three levels HC, NPSLE and non-NPSLE) and time between cognitive evaluations (in months) were entered both as fixed effects and as interaction effect. Subjects were included in the model as random effects. Normality across all groups and time points combinations was assessed statistically by means of Shapiro–Wilk tests and visually with Q-Q plots. This indicated deviations from normality across all groups and time points for the *simple attention* domain, as well as on the *complex attention* domain for non-NPSLE at baseline and NPSLE at follow-up. Among non-NPSLE, *visual memory* scores were non-normally distributed at baseline and *verbal memory* scores at follow-up.

## Results

### Cross-sectional analysis (at follow-up)

According to the CNS-VS interpretation guide, the overall cognitive performance was normal to high in 97% of HC, 69% of non-NPSLE and 76% of NPSLE patients. Based on adjusted *p* values, significant differences in mean performance as a function of group were observed for the domains *complex attention, psychomotor speed* and *motor speed*. Mean performance on the remaining cognitive domains did not differ significantly between the groups.

**Fig. 1:**
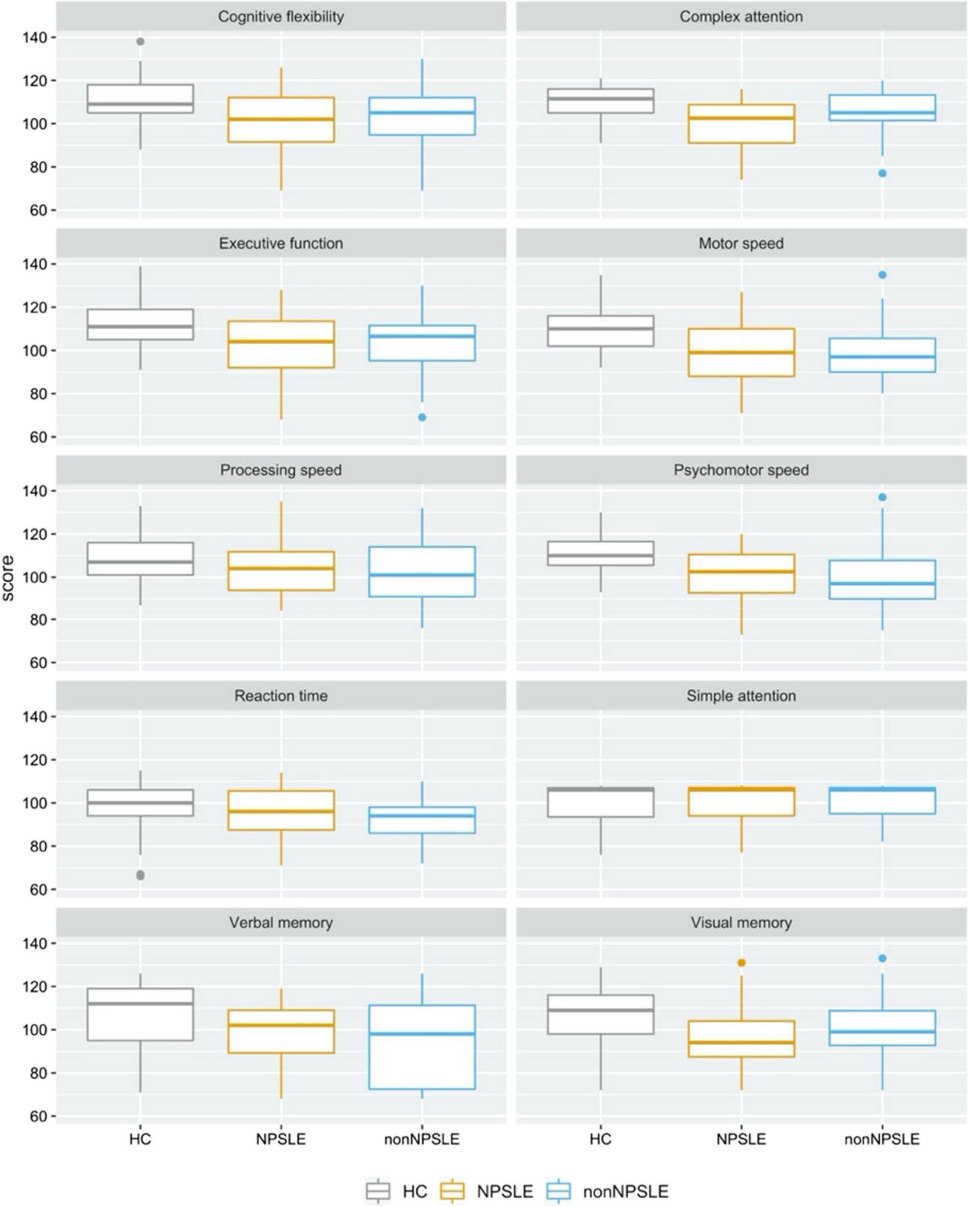
Distribution of standardized mean scores for NPSLE, non-NPSLE and HC for each cognitive domain at follow-up

Tukey’s test was computed to determine which groups differed significantly on the three cognitive domains for which the one-way ANOVA had revealed significant differences. The results suggest that HC and NPSLE differ significantly on all three domains. The scores of HC and non-NPSLE differ significantly on the domains *motor speed* and *psychomotor speed*, but not *complex attention*. The differences between NPSLE and non-NPSLE were not significant for any of the three domains. Details from the analysis can be found in Tables 3 and 4 and a graphical representation of how the scores were distributed in the three groups in all ten cognitive domains is presented in Fig. 1.

**Table 3:** Comparison of mean performance of NPSLE, non-NPSLE and HC on all cognitive domains at follow-up

Domain	F (df)	η_G_^2^
Cognitive flexibility	4.94 (2, 85)	0.10
Complex attention	7.73 (2, 83)**	0.16
Executive function	4.10 (2, 85)	0.09
Motor speed	7.64 (2, 86)**	0.15
Processing speed	1.69 (2, 87)	0.04
Psychomotor speed	7.63 (2, 84)^**^	0.15
Reaction time	1.63 (2, 83)	0.04
Simple attention	0.22 (2, 83)	0.01
Verbal memory	3.56 (2, 82)	0.08
Visual memory	3.05 (2, 83)	0.07

**Adj. *p* < .01

**Table 4:** Results from Tukey’s test for the cognitive domains for which significant group differences were found

Domain	Groups		Mean_Diff_	Simultaneous 95% CI
Complex attention	HC	NPSLE	− 10.72	[− 17.27, − 4.18]***
	HC	Non-NPSLE	− 4.54	[− 11.19, 2.12]
	NPSLE	Non-NPSLE	6.19	[− 0.36, 12.73]
Motor speed	HC	NPSLE	− 11.21	[− 18.92, − 3.49]**
	HC	Non-NPSLE	− 11.28	[− 19.39, − 3.17]**
	NPSLE	Non-NPSLE	− 0.07	[− 7.94, 7.79]
Psychomotor speed	HC	NPSLE	− 11.35	[− 20.41, − 4.29]**
	HC	Non-NPSLE	− 11.97	[− 20.40, − 3.53]**
	NPSLE	Non-NPSLE	− 0.62	[− 8.74, 7.50]

**Adj. *p* < .01. ***Adj. *p* < .001

### Clinical scores (at follow-up)

The values for organ damage and disease activity were overall quite low, with 81% of patients scoring 0 or 1 on the SLICC/ACR scale and 82% of patients scoring between 0 and 2 on the SLEDAI-2K scale.

### Association between cognitive performance and disease duration (at follow-up)

A correlational analysis (Pearson’s r) was performed to check for potential relationships between the ten cognitive domains and disease duration (in years). The individual correlation coefficients between the cognitive scores and disease duration ranged from 0.07 to 0.4 and were thus ranging somewhere between negligible and weak. The most noteworthy outcome from this analysis were weak positive correlations between disease duration and the *psychomotor speed* (*r* = 0.4), *motor speed* (*r* = 0.33) and *reaction time* (*r* = 0.33) scores. A summary detailing the correlational coefficients can be found in Table 5.

**Table 5:** Pearson’s correlation coefficients with 95% confidence intervals for the associations between cognitive scores and disease duration at follow-up

	Disease duration (in years)	95% CI
Psychomotor speed	0.4**	[0.4; 0.41]
Complex attention	0.08	[0.07; 0.09]
Visual memory	0.18	[0.17; 0.18]
Verbal memory	0.18	[0.17; 0.19]
Cognitive flexibility	0.07	[0.07; 0.08]
Executive function	0.07	[0.07; 0.08]
Processing speed	0.15	[0.14; 0.16]
Reaction time	0.33*	[0.32; 0.34]
Simple attention	0.25	[0.25; 0.26]
Motor speed	0.33*	[0.32; 0.34]

*p < .05. **p < .01

### Longitudinal analysis

**Fig. 2:**
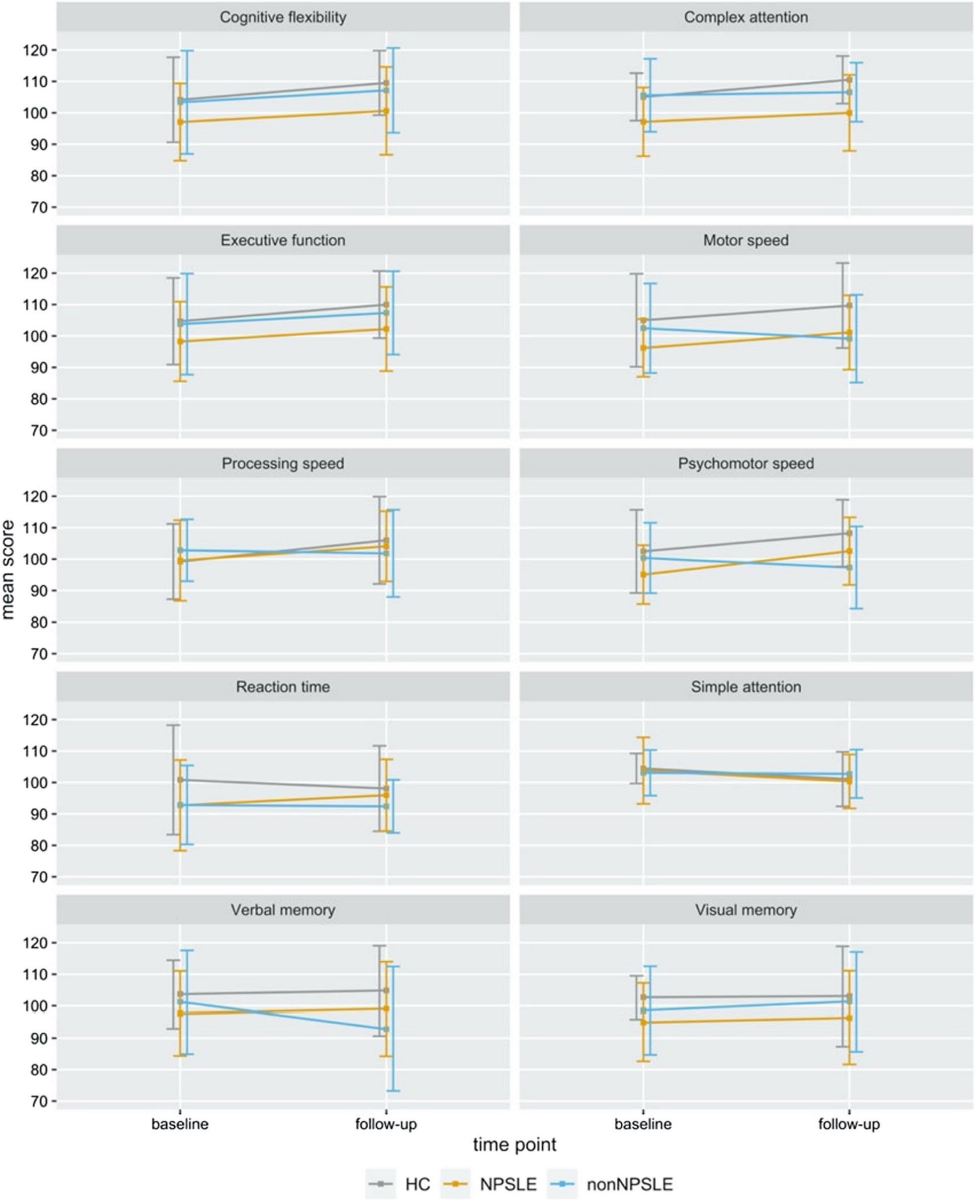
Development of group mean scores for each cognitive domain between baseline and follow-up. *Notes.* The error bars represent ± one standard deviation from the group mean

The repeated measures analyses indicated no significant effects of group (with the levels HC, NPSLE and non-NPSLE), time between baseline and follow-up assessment or an interaction between the two on any of the cognitive domains. A graphic overview of how the mean scores in each of the cognitive domains developed over time in three groups can be found in Fig. 2.

## Discussion

The primary objective with this study was to investigate the development of cognitive abilities in SLE patients over time. While the results do not point towards any temporal progression of cognitive performance, they do lend further support to previous research that found SLE patients to perform weaker in certain cognitive domains than the general population [11, 23]. Across all analyses, both longitudinal and cross-sectional, there were no significant performance differences between NPSLE and non-NPSLE patients – challenging the suitability of cognitive deficits as a distinguishing feature between patients with and without neuropsychiatric lupus. This result is somewhat at odds with earlier findings from our group and others, where significant differences in the cognitive performance of NPSLE and non-NPSLE patients were observed [11, 23].

### Cross-sectional findings

NPSLE and non-NPSLE patients’ performance on the domains *psychomotor speed* and *motor speed* was significantly lower than that of HC. Additionally, NPSLE patients’ scores were significantly lower than those of HC in *complex attention*. This is partially in line with an earlier study, which showed significant performance differences between NPSLE patients and HC in the domains *psychomotor speed* and *complex attention* [23]. It also coincides with a recent meta-analysis that found NPSLE, but not non-NPSLE patients, to perform significantly different from HC with respect to *complex attention* [11]. Supporting our findings further is the fact that according to the ACR, *psychomotor speed* and *complex attention* are two out of a total of five cognitive domains where significant deficits constitute cognitive dysfunction in NPSLE [7]. It is worth mentioning though, that unlike the current findings, our earlier significant results were uncorrected for multiple comparisons [23, 42, 46].

Previous research has revealed associations between the cognitive abilities of SLE patients and metrics of brain structure and function, such as altered functional connectivity or correlations between certain diffusion MRI metrics and cognitive performance [9, 12, 23, 24, 42, 46, 47]. The evidence is currently not conclusive, with some studies not finding any differences between non-NPSLE and NPSLE in terms of their white matter lesions or any relation between lesion load and cognitive performance [23, 46] while others did observe differences in white matter hyperintensities in NPSLE compared to non-NPSLE patients [48, 49]. While these studies have yielded some clues for possible neural underpinnings of cognitive dysfunction in SLE, these links are still diffuse and further research will be needed to specify how exactly cognitive impairment in SLE patients is manifested on the level of the brain.

There were some associations between patients’ cognitive scores and disease duration, namely weak positive correlations between patients’ performance on the *psychomotor speed*, *motor speed* and *reaction time* domain on one hand and disease duration on the other. While certainly counterintuitive at first glance, this finding can be viewed as in line with a previous study suggesting that longer disease duration lowered the risk of cognitive impairment [6], but could also indicate a skewed inclusion regarding disease duration in our study.

### Longitudinal findings

While the longitudinal data revealed significant group differences for the performance in *complex attention* at both time points, it did not confirm significant development over time in any of the cognitive domains. Several possible reasons for this lack of temporal effects can be noted; some of these have to do with the set-up of this study, others relate to the specific patient cohort studied. When it comes to design aspects of the study that might have influenced the results, it could be hypothesized that the time between the two neurocognitive examinations (mean 50 months) was not enough for a clear deterioration in cognitive performance among SLE patients to become apparent. This might be considered a limitation as it is currently unknown if 50 months is sufficient time to detect an effect on cognition in patients with SLE. However, the absence of cognitive decline over time in our sample is consistent with an earlier longitudinal study demonstrating no significant changes in the cognition of SLE patients, though it should be noted that the time between test sessions was substantially shorter than in the present study (approx. 18 months) [36].

Notably, the recruited patients had overall limited number and volume of white matter lesions, were clinically routinely followed adequately treated, and had low scores of disease activity. This might be a contributing factor to the lack of notable decline in cognitive abilities between baseline and follow-up assessment. As can be noted from Table 2 the SLEDAI-2K score were similar at both examinations. An explanation for this is that neuropsychiatric component of the SLEDAI-2K is very different from the definitions from ACR that were used (in addition to clinical examination) when determining presence of NPSLE or not. Thus, a patient may very well have a low SLEDAI-2K score and still display symptoms that merit an NPSLE diagnosis. Furthermore, the patients were consecutively included in the study, meaning that they were not included at a time point of new onset severe NPSLE, which most likely would have yielded a higher SLEDAI-2K at inclusion. In addition, patients were to a high extent on immunosuppressive treatment.

Also, noted is that only one patient converting from positive to negative in ANA and similarly for anti-Sm. We have avoided trying to draw any conclusions from one individual. Additionally, it is uncommon with seroconversion of ANA, and the same is true for anti-Sm. Overall in previous studies, becoming ANA negative is associated with a less severe disease.

Notably none of the NPLSE patients in the present study received cyclophosphamide (Table 2). This can be explained by the fact that the majority of patients were not included in the study because of new onset severe NPSLE. A few patients with severe manifestations requiring cyclophosphamide were not included until after the cyclophosphamide pulse regime were concluded.

### Limitations

The lack of longitudinal effects in the present sample may have arisen from varying test–retest intervals between participants, ranging from 19 to 78 months. Some factors limiting the statistical power of the study are the rather small sample size with a limited number of people in each group and for the longitudinal analysis the relatively unbalanced design, with 30 NPSLE, 22 non-NPSLE and 13 HC. The number of cognitive domains that were tested created a need to correct for multiple comparisons, which in turn further reduced statistical power. It should also be added that with less severe neuropsychiatric involvement, for example in the form of mild cognitive problems, it can be difficult to determine the extent to which it is related to SLE. The results of the present study might have looked different if a stricter classification system had been applied. The present analysis is based on a clinically well-controlled cohort of SLE patients that is overall doing well; documented for example by their very low SLEDAI-2K scores or the nature of their NPSLE manifestations. It should be noted however that a neurologist and a rheumatologist decided on a case-by-case basis whether symptoms were deemed to result from SLE or not; meaning that some patients suffered from for example headaches, anxiety, or depression, but that these were not automatically classified as an NPSLE manifestation. This might, at least in part, explain why we did not see any differences between NPSLE and non-NPSLE patients in this sample.

The current analysis did not take the participants’ educational level into account. It can only be speculated if lack of this information may have influenced the findings. Not all participants that were included at baseline, participated again at the follow-up examination. The examinations were performed during daytime work hours, which may have prohibited some of the original participants from partaking in the follow-up assessment.

### Future directions

Future investigations of cognition in SLE patients would benefit from following larger cohorts over an extended timeframe, with regular and ideally frequent cognitive re-evaluations. This way, it would be possible to get a more detailed picture of the longitudinal course of cognitive impairment in SLE patients, including potential fluctuations over time, presumably associated with other variables, such as disease activity, medication, mood, fatigue or indices of brain structure and function. Moreover, it could be worthwhile to test cognitive abilities in a more targeted way than has been the case. Instead of assessing a broad range of cognitive abilities, focusing on a few selected domains that have consistently emerged as heavily affected in previous research would help define the exact nature of the relationship between SLE and cognitive dysfunction.

## Conclusion

Cognitive dysfunction is a problem in SLE, both in patients with and without neuropsychiatric involvement. Some deviations in terms of the specific cognitive domains that are primarily affected in NPSLE and non-NPSLE patients notwithstanding, the findings of this study are overall in line with existent literature, which found cognitive deficits to be a reliable manifestation in the population of SLE patients as a whole [8, 11]. No significant progression of cognitive impairment over time was observed in SLE patients. This could be owed to the timeframe of the study, but it might also be an indication of a more complex relationship between SLE patients’ cognition and time than a purely linear one. In the future, further longitudinal examinations of the cognitive abilities of SLE patients will be essential to help the field gain a more refined understanding of how cognitive dysfunction develops in these patients over time.

## Supplementary Information

**Additional file 1.** Additional information regarding disease manifestations and age.

## Data Availability

The dataset generated and analyzed for the current study cannot be made publicly available, as this would violate Swedish law. The research was performed under an IRB approval and required the research participant to sign an informed consent. According to Swedish law applicable to this study, the scope of the consent must be specific (Personal Data Act 1998:204; Swe. “Personuppgiftslagen”, http://rkrattsbaser.gov.se/sfst?bet=1998:204). Therefore, we are prohibited from sharing the data publicly for general research that was not described in the consent form. Data are available upon request from researchers who have ethical approval from the corresponding author on reasonable request.

## Abbreviations

SLE: Systemic lupus erythematosus
NPSLE: Systemic lupus erythematosus with neuropsychiatric manifestations
Non-NPSLE: Systemic lupus erythematosus without neuropsychiatric manifestations
HC: Healthy control
ANAM: Automated neuropsychological assessment metrics
CANTAB: Cambridge neuropsychological test automated battery
CNS-VS: CNS-vital signs
SD: Standard deviation
SDI: Systemic Lupus International Collaborating/Clinical/ACR Organ Damage Index
SLEDAI-2k: Systemic Lupus Erythematosus disease activity index 2000
MMF: Mycophenolate mofetil
IVIG: Intravenous immunoglobulin
MRI: Magnetic resonance imaging
