# Supplementary material for: Cognitive performance in systemic lupus erythematosus patients: a cross-sectional and longitudinal study
Source: BMC Rheumatol. 2022 Apr 20;6:22. doi: 10.1186/s41927-022-00253-3 (PMC9019974; doi:10.1186/s41927-022-00253-3)
Supplement: Supplementary file 1 — Additional file 1. Additional information regarding disease manifestations and age. [file 41927_2022_253_MOESM1_ESM.docx]

**Additional file 1**

Additional information regarding disease manifestations and age.

**Table 1**

Number of the neuropsychiatric manifestations for the cross-sectional sample (at follow-up). Patients can have more than one manifestation. The manifestations are according to the American College of Rheumatology case definitions for neuropsychiatric systemic lupus erythematosus.

|  | **nonNPSLE** | **NPSLE** |
| --- | --- | --- |
| *No. of subjects* | 29 | 33 |
| *Acute confusion* | 0 | 3 |
| *Anxiety* | 2 | 10 |
| *Aseptic meningitis* | 1 | 1 |
| *Cerebrovascular disease* | 0 | 3 |
| *Chorea* | 0 | 1 |
| *Cognitive dysfunction* | 6 | 19 |
| *Demyelinating disease* | 0 | 3 |
| *Depression* | 9 | 15 |
| *Epileptic seizures* | 0 | 1 |
| *Headache* | 11 | 22 |
| *Myelitis* | 0 | 2 |
| *Psychosis* | 0 | 2 |
| *Neuropathy (autonomic)* | 1 | 9 |
| *Neuropathy (cranial)* | *0* | *5* |
| *Guillain-Barre* | *0* | *0* |
| *Mononeuritis* | *0* | *2* |
| *Myasthenia gravis* | *0* | *0* |
| *Plexopathy* | *0* | *1* |
| *Polyneuropathy* | *0* | *2* |
|  |  |  |

**Additional file 1**

**Table 2**

Number of the neuropsychiatric manifestations at baseline and follow-up for the longitudinal sample. Patients can have more than one manifestation. The manifestations are according to the American College of Rheumatology case definitions for neuropsychiatric systemic lupus erythematosus.

|  | **Baseline** | | **Follow-up** | |
| --- | --- | --- | --- | --- |
|  | nonNPSLE | NPSLE | nonNPSLE | NPSLE |
| *No. of subjects* | 22 | 30 | 22 | 30 |
| *Acute confusion* | 0 | 3 | 0 | 3 |
| *Anxiety* | 3 | 11 | 2 | 8 |
| *Aseptic meningitis* | 1 | 1 | 1 | 1 |
| *Cerebrovascular disease* | 0 | 3 | 0 | 3 |
| *Chorea* | 0 | 2 | 0 | 1 |
| *Cognitive dysfunction* | 3 | 17 | 6 | 17 |
| *Demyelinating disease* | 0 | 3 | 0 | 3 |
| *Depression* | 9 | 17 | 8 | 12 |
| *Epileptic seizures* | 1 | 1 | 0 | 1 |
| *Headache* | 9 | 27 | 9 | 20 |
| *Myelitis* | 0 | 2 | 0 | 2 |
| *Psychosis* | 0 | 2 | 0 | 2 |
| *Neuropathy (autonomic)* | 1 | 8 | 1 | 9 |
| *Neuropathy (cranial)* | 0 | 6 | 0 | 5 |
| *Guillain-Barre* | 0 | 0 | 0 | 0 |
| *Mononeuritis* | 0 | 2 | 0 | 2 |
| *Myasthenia gravis* | 0 | 0 | 0 | 0 |
| *Plexopathy* | 0 | 2 | 0 | 1 |
| *Polyneuropathy* | 0 | 3 | 0 | 2 |

**Testing for age differences between the groups**

*Cross-sectional (N=91)*: A Kruskal-Wallis test suggested no significant age differences between the three groups.

*Longitudinal (N=65)*: A Kruskal-Wallis test and subsequent pairwise comparison indicated significant differences in age between HC and non-NPSLE as well as between NPSLE and non-NPSLE. However, these differences disappeared when adjusted for multiple comparisons.
